# Soil Mineral Nitrogen Dynamics and Yield Stability in Maize Under Variable Precipitation: Comparing Leguminous Green Manures with Mineral Nitrogen Fertilization in a Two-Year Pilot Study

**DOI:** 10.3390/plants15162424

**Published:** 2026-08-08

**Authors:** László Zsombik, Tibor József Aranyos, Csaba Juhász, Tamás Sipos, Tamás Magyar, Waleed A. E. Abido, Vivien Pál

**Affiliations:** 1 Research Institute of Nyíregyháza, Institutes for Agricultural Research and Educational Farm, University of Debrecen, 4400 Nyíregyháza, Hungary; zsombik@agr.unideb.hu (L.Z.); aranyos.tibor@agr.unideb.hu (T.J.A.); juhasz.csaba@agr.unideb.hu (C.J.); sipost@agr.unideb.hu (T.S.); 2 Institute of Water and Environmental Management, Faculty of Agricultural and Food Sciences and Environmental Management, University of Debrecen, 4032 Debrecen, Hungary; magyar.tamas@agr.unideb.hu; 3 Agronomy Department, Faculty of Agriculture, Mansoura University, Mansoura 35516, Egypt; madawy78@mans.edu.eg

**Keywords:** green manure, nitrogen dynamics, fertilization, soil mineral nitrogen, nutrient management, maize, sandy soil

## Abstract

Regenerative farming practices, including the use of leguminous green manures, are increasingly recognized for their potential to improve soil quality, enhance nutrient cycling, and reduce nitrogen losses. In this study, we investigated the effects of two leguminous green manure crops, lupine (*Lupinus albus* L.) and common vetch (*Vicia sativa* L.), on maize (*Zea mays* L.) yield and soil mineral nitrogen dynamics in two soil layers (0–25 cm and 25–50 cm). The treatments were compared with mineral nitrogen fertilization (80 kg ha^−1^ N) and an unfertilized control under field conditions on humic sandy soil in Hungary between 2021 and 2023. The results showed that under extreme drought conditions (2022), maize yield in mineral-fertilized plots (0.90 t ha^−1^) was significantly lower than in the unfertilized control (1.61 t ha^−1^), while green manure treatments maintained substantially higher yields (4.11–4.21 t ha^−1^). Soil nitrogen dynamics were strongly influenced by precipitation patterns. Correlation and regression analyses revealed that increasing precipitation reduced mineral nitrogen content in the topsoil, while promoting its accumulation in deeper soil layers. This effect was particularly pronounced in mineral-fertilized treatments, where a significant negative correlation was observed in the 0–25 cm layer and a positive correlation in the 25–50 cm layer, suggesting greater downward movement of mineral nitrogen within the soil profile. Principal component analysis further indicated that mineral fertilizer-derived nitrogen was more strongly associated with precipitation variables than nitrogen originating from green manure treatments. The results suggest that the application of leguminous green manures promotes a more stable soil nitrogen distribution and reduces sensitivity to precipitation-driven nitrogen redistribution compared with mineral fertilization.

## 1. Introduction

Improving water-use efficiency and reducing the dependence of maize production on mineral nitrogen fertilizers are important objectives of sustainable crop production, especially under increasingly variable climatic conditions. Sustainable crop production relies on efficient soil and nutrient management, including the rational use of water and nutrient resources and the conservation or enhancement of soil organic matter, to which green manuring makes an important contribution.

Climate change has increased the frequency of weather extremes, posing major challenges for crop production. In Hungary, the uneven temporal and spatial distribution of precipitation and the rising frequency of hot days increasingly require new agricultural practices [1]. The use of crop rotation systems greatly improves the efficiency of crop production by reducing negative stressors compared to monoculture cropping systems [2,3,4].

Crop rotation-based management systems can influence the type, extent and severity of soil degradation through their effects on organic carbon stocks, soil structure, water and nutrient management properties and nitrate leaching [5,6,7,8]. Diversified cropping systems form the basis of sustainable nutrient management, with green manures and cover crops playing a key role in maintaining soil fertility and crop productivity [9].

The lack of organic fertilizers, the rising fertilizer costs, and the physical and biological degradation of soils require the re-introduction of green manure crops as main and secondary crops [10]. Incorporating green manure crops into crop rotations improves soil water management, increases soil organic matter content, and reduces damage caused by water and wind erosion [11]. To maximize biomass, nitrogen uptake and soil cover, it is recommended that second crops be sown in the early months of the growing season, typically August to September [6] under continental climatic conditions. In addition to sowing date, pre-sowing precipitation and mean growing-season temperature are the main factors determining the above-ground biomass production of green manure crops [12,13]. Several authors have suggested that using green manures with nitrogen-fixing plant species should be considered as an alternative to fallowing to improve degraded soils [9,14,15]. The yield and quality of crops grown after green manure crops are influenced by various factors. These include the species, variety and biomass yield of the green manure crop; the C:N ratio; the date of organic matter incorporation into the soil; and the growing site, soil, and weather conditions [12].

Sandy soils are characterized by low organic matter content, poor nutrient retention capacity and a high susceptibility to nitrate leaching, particularly under intensive rainfall events [16]. Consequently, optimizing nitrogen release from green manure crops under these conditions is particularly important for improving nitrogen use efficiency and reducing environmental losses.

Leguminous green manures, such as lupine (*Lupinus albus* L.) and common vetch (*Vicia sativa* L.) represent a sustainable alternative to synthetic nitrogen fertilizers in arable cropping systems. Through symbiotic nitrogen fixation with Rhizobium bacteria, these species enrich the soil with plant-available nitrogen while simultaneously increasing soil organic matter, stimulating microbial activity, improving soil structure, enhancing water retention, and reducing nitrate leaching and soil erosion [13,17,18,19,20]. Furthermore, when incorporated into crop rotations, legumes help break pest and disease cycles, suppress weeds, and enhance agroecosystem resilience [21,22].

Several studies have demonstrated that leguminous cover crops integrated into maize-based cropping systems can fix between 50 and 200 kg N ha^−1^ [23], thereby increasing the plant-available nitrogen content of the soil, and supporting maize yields comparable to those achieved with moderate rates of synthetic fertilization [24,25,26,27]. Biologically fixed nitrogen can substantially reduce the requirement for inorganic fertilizers while maintaining or increasing crop productivity [28]. The root systems of green manure crops have been shown to improve soil nutrient and water cycling, thereby creating optimal conditions for subsequent crop growth [29] and reducing the following crop’s dependence on inorganic nitrogen fertilizer [30]. The use of legume green manures maintains soil fertility and biological activity [31], and their application can reduce the amount of nitrogen fertilizer required [32]. The relatively narrow C:N ratio (10–15) promotes rapid residue decomposition and nitrogen mineralization following incorporation, ensuring an efficient nitrogen supply during the early stages of maize development [33,34].

Among leguminous green manure species, lupines (*Lupinus* spp.) are particularly valuable for sandy soils because of their deep rooting system, high biomass production and ability to improve soil structure while increasing soil nitrogen and phosphorus availability [35,36,37]. In Hungary, the most widespread are white lupine (*Lupinus albus* L.), yellow lupine (*Lupinus luteus* L.), and blue lupine (*Lupinus angustifolius* L.).

Common vetch (*Vicia sativa* L.) is widely used as a green manure because of its high biomass production, rapid residue decomposition and efficient biological nitrogen fixation [38,39,40]. Previous studies have shown that incorporation of common vetch significantly increases soil mineral nitrogen availability, improves maize growth and yield, and enhances crop physiological performance, confirming its value as an effective green manure in maize-based cropping systems [41,42,43,44].

Collectively, these findings demonstrate that leguminous green manures have considerable potential to replace part of the mineral nitrogen fertilizer requirement while improving soil fertility and crop productivity. Although the positive effects of leguminous green manure on crop yield and soil fertility are well documented, less information is available on how the temporal and vertical distribution of green manure and mineral fertilizer nitrogen differs in sandy soils under different precipitation conditions. This study is particularly relevant to the sandy soils of Hungary and Central Europe, where low organic matter content and frequent summer droughts limit nitrogen retention and crop productivity. However, as a two-year pilot study, it provides preliminary evidence and was not designed to isolate the effects of individual climatic variables.

To address this knowledge gap, the present study evaluated the effects of leguminous green manures and mineral nitrogen fertilization on soil mineral nitrogen dynamics and maize performance under contrasting precipitation conditions. The specific objectives were as follows: (i) to compare the effects of leguminous green manure crops (*Lupinus albus* L. and *Vicia sativa* L.) and mineral nitrogen fertilization on soil mineral nitrogen (NO_2_^−^-N + NO_3_^−^-N) dynamics in a maize-based cropping system; (ii) to evaluate the temporal and vertical distribution of soil mineral nitrogen in two soil layers (0–25 cm and 25–50 cm) across two growing seasons with contrasting precipitation conditions; and (iii) to assess the preceding-crop effects of leguminous green manures in terms of maize growth and grain yield.

Based on previous findings, we had the following hypotheses: (H1) Leguminous green manures increase mineral nitrogen availability in the soil compared to the unfertilized control treatment; (H2) Nitrogen from mineral fertilizers is more strongly affected by precipitation-related soil profile redistribution than nitrogen from leguminous green manures; and (H3) Leguminous green manures improve maize grain yields comparable to those achieved with mineral nitrogen fertilization across two growing seasons with contrasting precipitation conditions.

## 2. Materials and Methods

### 2.1. Experimental Site Description

**Figure 1:**
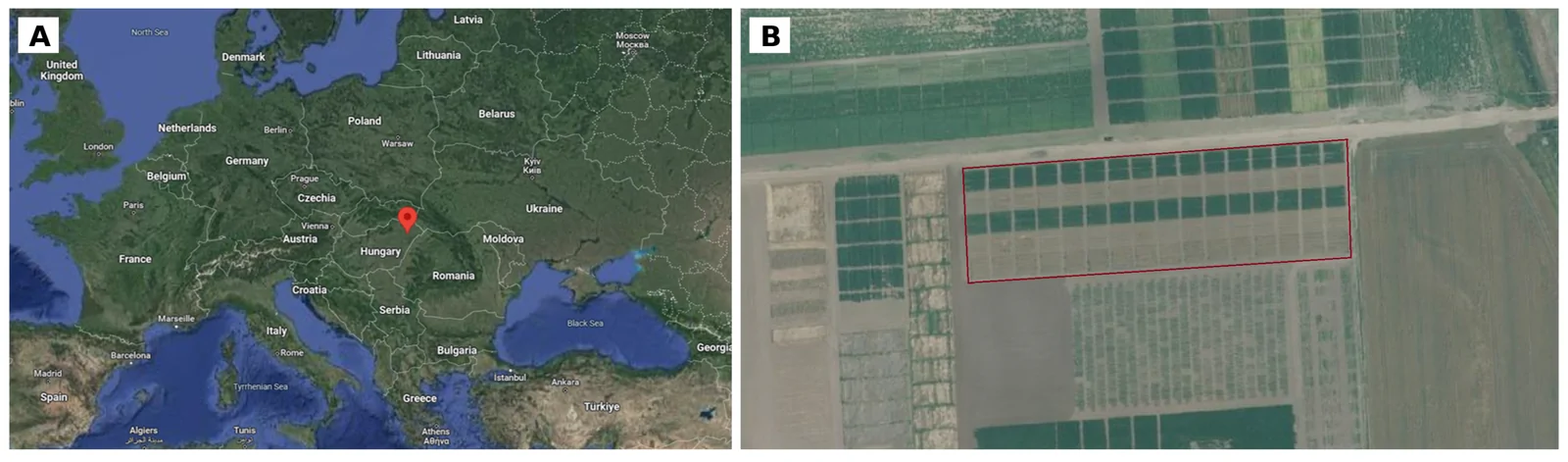
Location map of the experimental site and aerial view of the field experiment. (**A**) Location of Nyíregyháza in Hungary. (**B**) Aerial image of the experimental field at the UD IAREF Research Institute of Nyíregyháza. The red outline indicates the area of the crop rotation experiment.

The crop rotation experiment was set up at the University of Debrecen IAREF Research Institute of Nyíregyháza, Hungary (47.976272° N, 21.697241° E, Figure 1), to investigate the effect of two leguminous plants (lupine—*Lupinus albus* L., common vetch—*Vicia sativa* L.) on soil nitrite + nitrate nitrogen content, and the green manure value in terms of maize yield, compared with mineral-fertilized (80 kg ha^−1^ N) and control treatments.

The experimental site is located on a humic sandy soil classified as a Humic Arenosol according to the WRB (World Reference Base for Soil Resources) classification system. The soil texture was characterized by high sand content (87.11%), with 3.66% silt and 9.43% clay. General soil chemical properties were determined in August 2021 at the beginning of the evaluation period covered by the present study. Eight soil samples were collected from each crop rotation area (Crop Rotation I and II) at two soil depths (0–25 and 25–50 cm) and analyzed in an accredited soil laboratory. Table 1 summarizes the general soil chemical properties of the two crop rotation areas, including soil pH, carbonate lime, organic matter, mineral nitrogen (NO_3_^−^-N + NO_2_^−^-N), available K_2_O and available P_2_O_5_. Differences between crop rotations and soil depths were evaluated using two-way ANOVA.

### 2.2. Precipitation and Temperature Characteristics Observed During the Research Period

**Figure 2:**
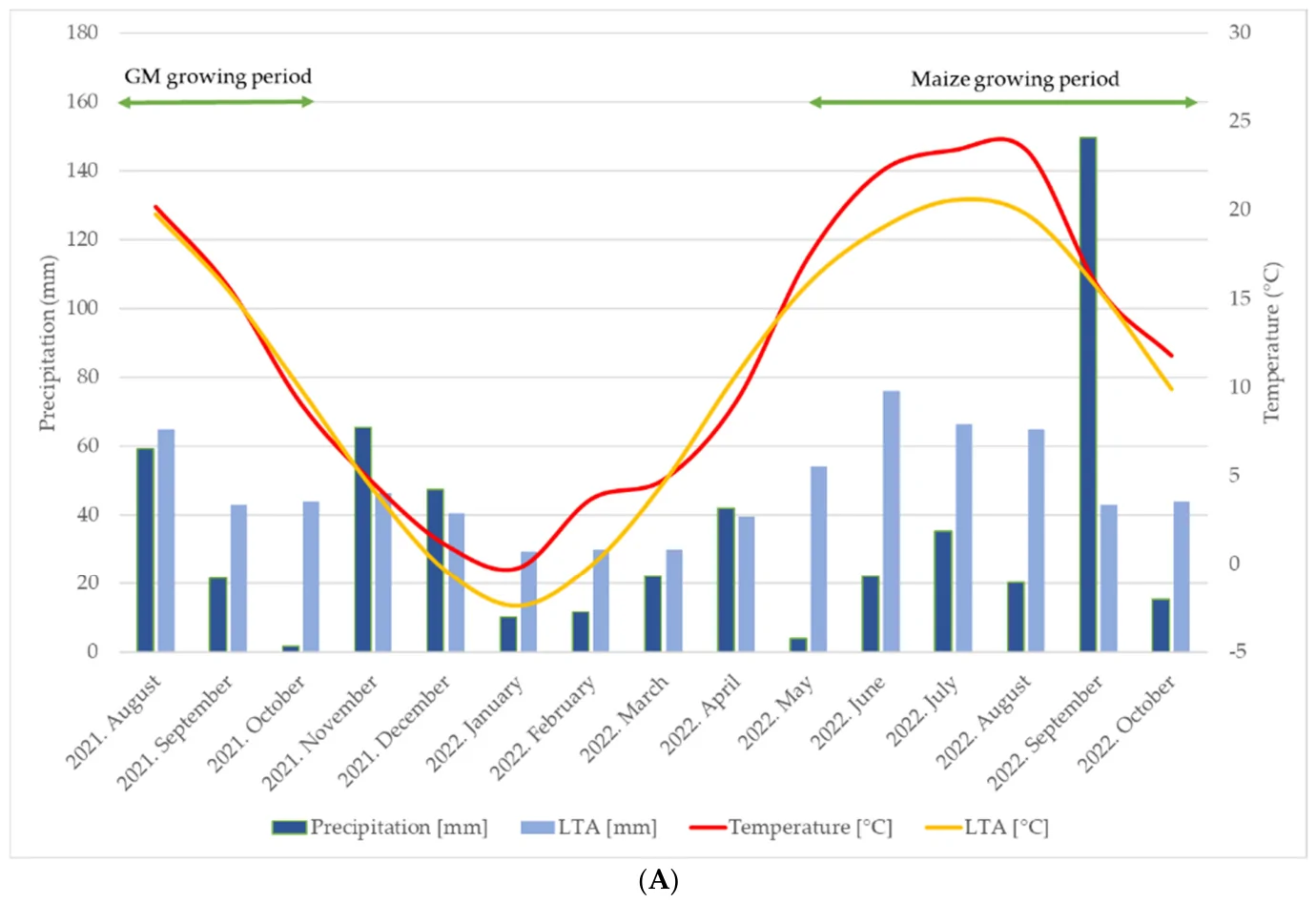

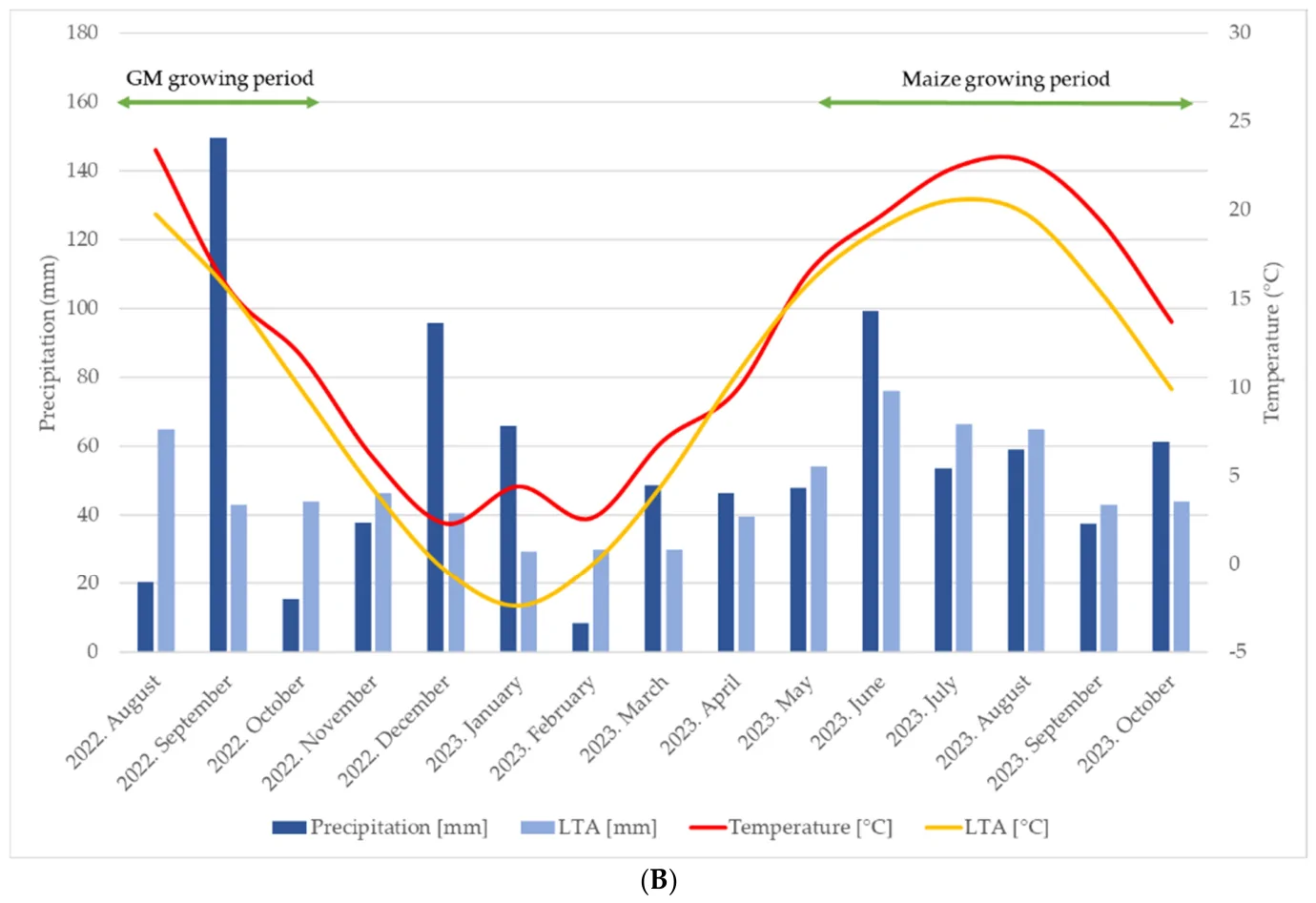
Long-term average (LTA) and total monthly precipitation and average monthly air temperature for years (**A**) 2021–2022 and (**B**) 2022–2023. Bars and left y-axis represent precipitation, and right y-axis and lines represent temperature data. GM: Green manure.

Meteorological data (precipitation, temperature) were measured using a μMETOS weather station (Pessl Instruments GmbH, Weiz, Austria). Figure 2 demonstrates the precipitation and temperature conditions for the years studied, with averages over the last 30 years (LTA), and indicates the growing periods of green manures (GM) and maize.

In 2021, precipitation levels were particularly low, with 21.5 mm of rain recorded in September and 1.7 mm in October. These conditions were conducive to the unfavorable development of green manure plants. The 2022 crop year was characterized by extreme precipitation patterns. The annual rainfall was 98.3 mm below the 30-year average, and the rainfall distribution was highly uneven. From January to August, with the exception of April, the monthly rainfall was below the average, resulting in a water deficit of 223.3 mm. The prevailing drought resulted in an extremely stressful situation for maize. In September, 149.6 mm of rainfall was recorded, 106.6 mm above average. For the green manures, the rainfall in September permitted vegetative development. With regard to temperature, the months of June, July and August were 2.8–3.6 °C above average, combined with a lack of rainfall. The precipitation recorded in 2023 was 590 mm, which is 26 mm above the 30-year average. In the period following the 2022 drought, precipitation was evenly distributed, with spring and summer amounts approximating average values. Temperatures during the winter months were above average, but April, May, and June were below average. Temperatures in August, September, and October were all above average, which, combined with sufficient rainfall, provided optimal conditions for the development of green manure plants.

### 2.3. Experimental Design

**Figure 3:**
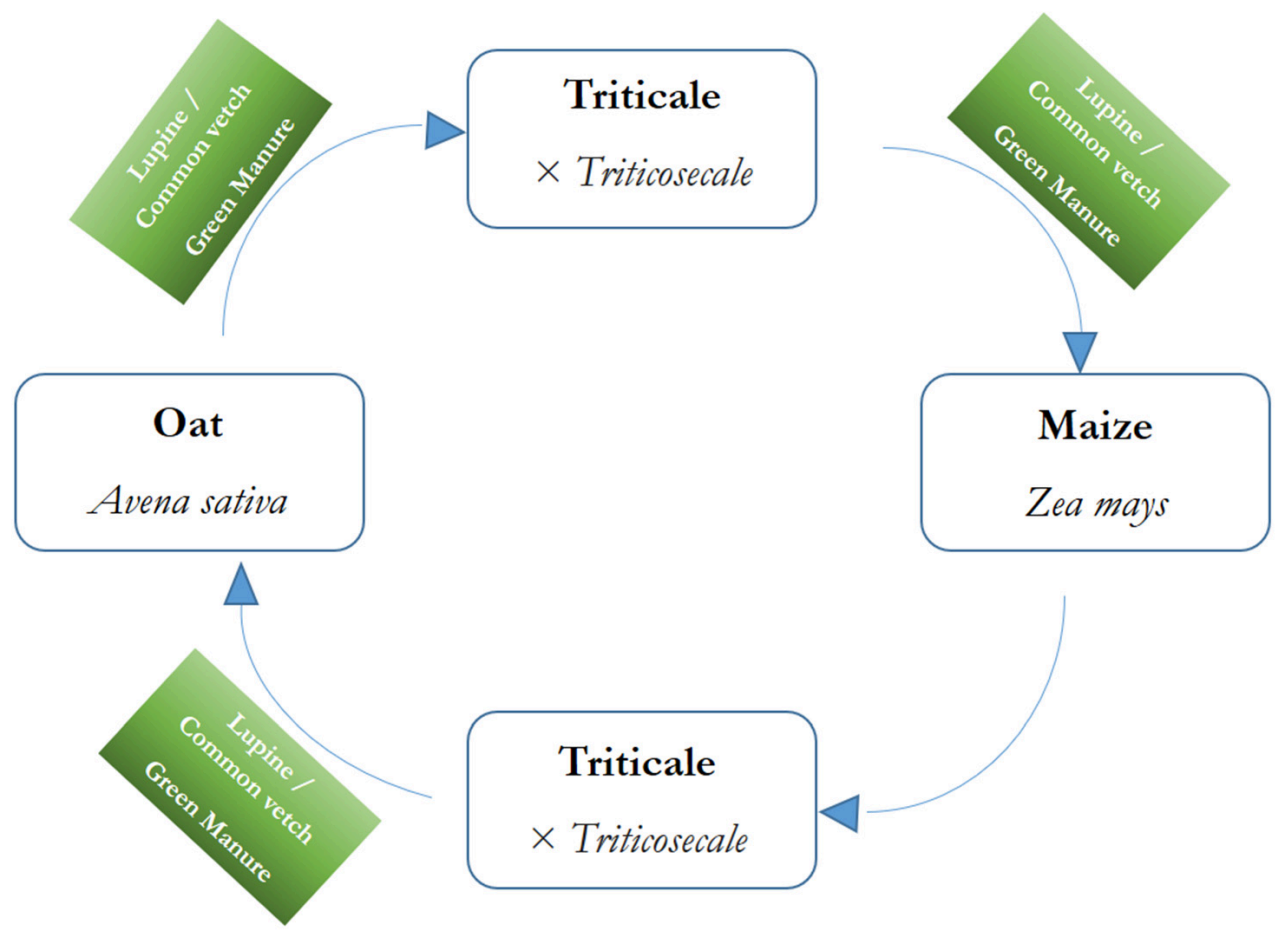
Crop sequence in the crop rotations and the schedule of green manure application.

**Figure 4:**
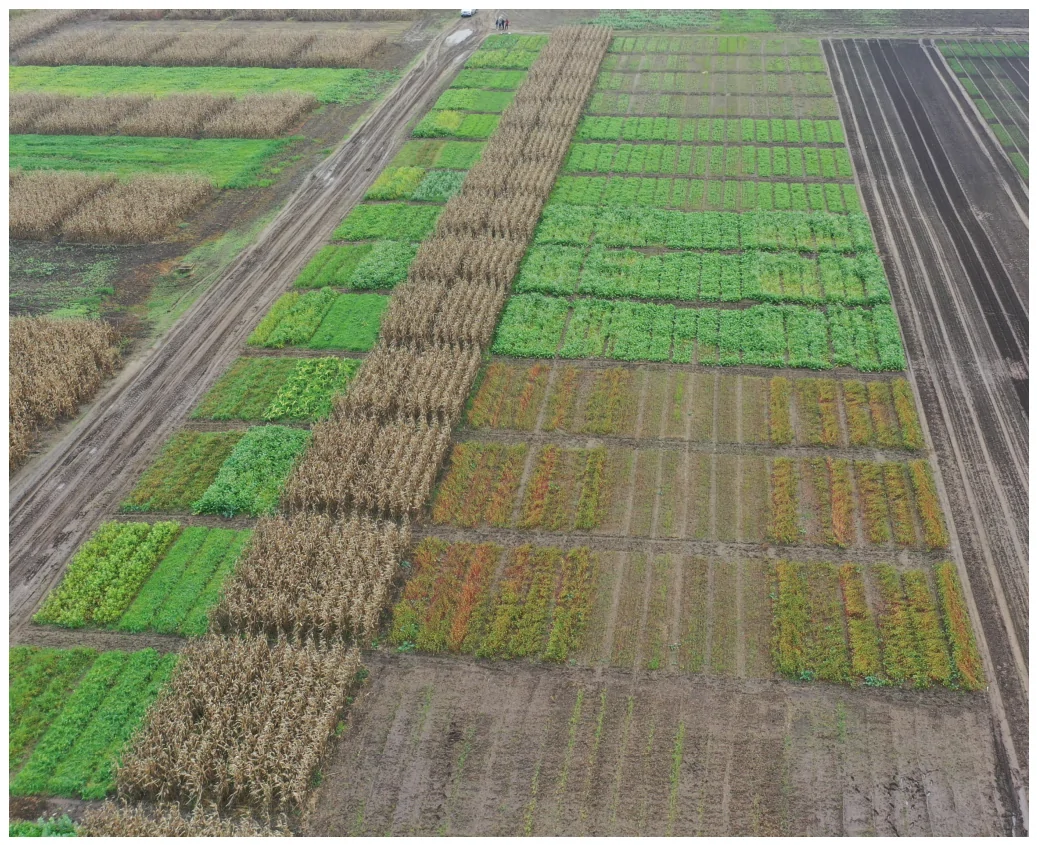
View of the crop rotation experiment in October.

The crop rotation experiment was established in 2019 and includes maize, triticale, and oat as the main crops (Figure 3 and Figure 4). The long-term crop rotation experiment consists of four parallel crop rotation systems. The present study focuses on Crop Rotations I and II and evaluates soil mineral nitrogen dynamics from autumn 2021 to autumn 2023, together with maize yield in the 2022 and 2023 growing seasons. The experiment investigates the effects of two leguminous green manure plants—white lupine (*Lupinus albus* L.) and common vetch (*Vicia sativa* L.)—on soil properties and the yield of subsequent crops. The present study evaluates the impact of these leguminous green manure plants on soil nitrite and nitrate nitrogen content and maize yield. The effect of green manure treatments is compared with those of the mineral-fertilized and control treatments. The fertilizer treatments were applied during the maize growing season, at the 4–6 leaf stage, while the inter-row cultivator was being used. Fertilizer was applied in the form of calcium ammonium nitrate at a rate of 80 kg ha^−1^ of active nitrogen. No phosphorus or potassium fertilizers were applied in any treatment during the study period. In the case of the fertilization treatment, no green manure was grown. In the green-manured plots, no fertilizer was applied during the entire experiment. In the control treatment, neither green manure nor fertilizer was applied. During the green manure growing period, the mineral-fertilized and control plots were left fallow. The treatments were arranged in a randomized complete block design (RCBD) with four replications.

The experiment simultaneously evaluates two parallel crop rotation systems, enabling assessment of certain crop species over consecutive years. The sequence of crops within the rotations during the study period is illustrated in Table 2, providing the experimental context for the maize crops evaluated in 2022 and 2023 and for the baseline soil mineral nitrogen assessment conducted prior to the first green manure sowing. Sowing of the green manure species was carried out in early August, following the harvest of cereal crops, and termination occurred at the end of October. All treatments were applied to the same plots each year, allowing for cumulative effects over time.

Prior to the first green manure sowing (10 August 2020), baseline soil samples were collected from all experimental plots. At that time, no treatments had yet been applied. The samples were taken from the plots subsequently assigned to the lupine, common vetch, mineral-fertilized and control treatments, allowing verification of the initial homogeneity of the experimental units before treatment application. Following baseline sampling, the plots were assigned to the experimental treatments according to a randomized complete block design, and the same plots were maintained throughout the experiment. Although the experiment consisted of four field replications, soil mineral nitrogen was consistently monitored in the same three replicate plots per treatment from the baseline sampling onward and throughout the entire study period. Crop Rotations I and II differed in their preceding cereal crops (triticale and oat, respectively); therefore, baseline soil mineral nitrogen data are presented separately for each crop rotation (Table 3). Baseline mineral N data were subsequently evaluated using a factorial repeated-measures ANOVA, with crop rotation and future treatment assignment as between-subject factors and soil depth as the within-subject factor. No significant effect of future treatment assignment or treatment-related interactions was detected (*p* > 0.05), confirming that the plots allocated to the four treatments were comparable before treatment establishment.

### 2.4. Agrotechnical Data and Measurements

Green manure crops were sown using a Wintersteiger Plot Spider plot seeder (Wintersteiger Seedmech GmbH, Ried im Innkreis, Austria), while maize was sown with a Monosem NG+ seed driller (Ribouleau Monosem, Largeasse, France). The cash crops were harvested using a Zürn SE 130 plot combine harvester (Zürn Harvesting GmbH & Co. KG, Waldenburg, Germany).

The gross plot size was 1.7 × 9.2 m (15.64 m^2^). Soil preparation and plant protection operations followed conventional crop production practices. The seed density for the common vetch green manure was 2.5 million seeds ha^−1^, and for lupine, 0.5 million seeds ha^−1^. The 1000-seed weight ranged from 31.5 to 46.5 g for common vetch and from 285 to 310 g for lupine. So, common vetch requires 3910 seeds plot^−1^, resulting in a mass of 123.2–181.8 g, while lupine requires 782 seeds plot^−1^, resulting in a mass of 222.9–242.4 g. Green manure crops were sown on 3 August and 12 August, and were terminated following intensive vegetative development—at flowering time—between 27 October and 28 October (Table 4). During termination, the green manure biomass was crushed above the soil surface and then incorporated into the soil through ploughing at a depth of 30 cm.

Prior to termination at the end of October, the above-ground biomass yield of the green manures was assessed by using a 50 × 50 cm sampling frame placed in each replication. Above-ground biomass was harvested from a 0.25 m^2^ area and weighed to determine green yield.

Maize (*Zea mays* L. cv. DKC 4316) was sown on 3 May in both investigated years with a seed density of 65,000 seed ha^−1^. Plant height was recorded at the end of the flowering stage after 64 days from sowing, using five replicates per plot. After harvest, the yield from each plot was measured.

To evaluate the effects of green manure on soil nitrogen content, soil samples were collected at three time points: following green manure incorporation, following maize sowing, and following maize harvest. Samples were taken from two soil depths (0–25 cm and 25–50 cm) using a hammer-driven soil sampler with a 5 cm diameter core. The sampling depths reflected the tillage system applied in the experiment. The experimental field was mouldboard-ploughed to approximately 30 cm; therefore, the incorporated green manure biomass was distributed throughout the cultivated soil layer rather than remaining only in the uppermost soil. Consequently, the 0–25 cm layer represented the main cultivated zone, whereas the 25–50 cm layer allowed the assessment of mineral nitrogen dynamics immediately below the plough layer and the potential downward movement of mineral nitrogen in the sandy soil. The field experiment was established with four replications; soil sampling was consistently conducted in the same three replicate plots for each treatment throughout the study period, ensuring comparability across sampling dates. The samples were analyzed in an accredited laboratory using a Thermo Scientific Gallery Discrete Analyzer (Thermo Fisher Scientific Oy, Vantaa, Finland) to determine the concentrations of nitrite and nitrate nitrogen.

### 2.5. Statistical Analysis

Statistical analyses were performed using IBM SPSS Statistics (version 26, IBM Corp., Armonk, NY, USA). Prior to statistical analyses, the assumptions of normality and homogeneity of variances were evaluated. Statistical significance was accepted at *p* < 0.05.

General soil chemical properties determined at the beginning of the evaluation period (2021) were analyzed using factorial repeated-measures ANOVA, with crop rotation as the between-subject factor and soil depth (0–25 cm and 25–50 cm) as the within-subject factor.

Baseline soil mineral nitrogen determined prior to the first green manure sowing (2020) was further evaluated using factorial repeated-measures ANOVA, with crop rotation and future treatment assignment as between-subject factors and soil depth as the within-subject factor to verify the initial comparability of the experimental plots before treatment establishment.

Above-ground fresh biomass of the green manure crops, maize plant height and maize grain yield were analyzed using two-way analysis of variance (ANOVA), with growing season and legume species (green manure biomass) or nutrient management strategy (plant height and grain yield) as fixed factors. Main effects and interactions were evaluated, and descriptive statistics were presented as mean ± standard deviation (SD).

Soil nitrite + nitrate nitrogen content was analyzed separately for the 0–25 cm and 25–50 cm soil layers using repeated-measures ANOVA, with growing season and nutrient management strategy as between-subject factors and soil sampling date (following green manure incorporation, maize sowing and maize harvest) as the within-subject factor. Mauchly’s test was used to assess the assumption of sphericity. Where the assumption was met, the sphericity-assumed model was used for statistical inference.

Pearson correlation analysis was conducted to evaluate the relationships between precipitation variables and soil mineral nitrogen content at different soil depths under the various treatment conditions.

Linear regression analysis was performed to quantify the relationships between soil mineral nitrogen content and precipitation indicators, including cumulative precipitation (1, 3 and 6 months prior to sampling) and the number of days with precipitation exceeding 10 mm over the same periods. Coefficients of determination (R^2^) and significance levels (*p*-values) were used to assess the strength and significance of the relationships.

Principal component analysis (PCA) was conducted using Statgraphics Centurion 18 (version 18.1.12, Statgraphics Technologies, Inc., The Plains, VA, USA). Prior to analysis, all variables were standardized (z-scores) to eliminate the effect of differing measurement scales. Components with eigenvalues greater than 1 were retained according to the Kaiser criterion. PCA results were interpreted based on component loadings and visualized using biplots.

## 3. Results

### 3.1. Effect of Precipitation and Crop Year on the Above-Ground Fresh Biomass Weight of Green Manures

Weather conditions during the green manure growing period differed markedly between the two experimental years, particularly with respect to precipitation in September and October. In September and October 2021, precipitation totaled 21.5 mm and 1.7 mm, respectively, whereas in 2022 the corresponding values were 149.6 mm and 15.4 mm. Accordingly, above-ground fresh biomass production was substantially higher in 2022 than in 2021 for both legume species (Table 5). White lupine (*Lupinus albus* L.) produced 0.41 ± 0.30 t ha^−1^ in 2021 and 3.04 ± 0.66 t ha^−1^ in 2022, while common vetch (*Vicia sativa* L.) produced 0.73 ± 0.11 and 2.97 ± 0.46 t ha^−1^, respectively. Two-way ANOVA revealed a significant effect of growing season on above-ground fresh biomass production (*p* < 0.001), whereas neither legume species (*p* = 0.567) nor the growing season × legume species interaction (*p* = 0.386) had a significant effect.

### 3.2. Effect of Green Manure Application on the Plant Height and Seed Yield of Maize

During the maize growing seasons of 2022 and 2023, plant height varied according to both weather conditions and nutrient management strategy. In the extreme drought year of 2022, maize plants grown after white lupine and common vetch green manures reached mean heights of 180.86 and 184.25 cm, respectively, whereas plant height under mineral N fertilization and the unfertilized control averaged 149.17 and 151.83 cm, respectively. In contrast, under the more favourable growing conditions in 2023, plant height was consistently high across all nutrient management strategies, ranging only from 304.00 to 307.44 cm. Two-way ANOVA revealed significant effects of growing season (*p* < 0.001) and nutrient management strategy (*p* = 0.002), as well as a significant growing season × nutrient management strategy interaction (*p* = 0.003), indicating that the effect of nutrient management strategy on maize plant height differed between the two growing seasons (Table 6; Supplementary Table S1A).

Maize grain yield varied markedly between the two growing seasons and among nutrient management strategies (Table 7). During the extreme drought of 2022, grain yields were substantially higher following white lupine and common vetch green manures (4.21 and 4.11 t ha^−1^, respectively) than under mineral N fertilization (0.90 t ha^−1^) or in the unfertilized control (1.61 t ha^−1^). In contrast, under the more favourable weather conditions of 2023, grain yield increased considerably across all nutrient management strategies, ranging from 9.45 to 11.71 t ha^−1^. Two-way ANOVA revealed significant effects of growing season (*p* < 0.001) and nutrient management strategy (*p* < 0.001), as well as a significant growing season × nutrient management strategy interaction (*p* < 0.001), indicating that the response of maize grain yield to nutrient management strategy depended on the growing season (Table 7; Supplementary Table S1B).

### 3.3. Effect of Green Manure Application on the Soil Nitrite + Nitrate N Content Compared with Fertilization and Control Treatments

Before evaluating the effects of green manure and mineral fertilization, the comparability of the experimental plots was verified using baseline soil mineral N data. Factorial repeated-measures ANOVA showed significant effects of crop rotation (*p* < 0.001) and soil depth (*p* < 0.001), as well as a significant crop rotation × soil depth interaction (*p* = 0.043). In contrast, neither future treatment assignment (*p* = 0.895) nor any interaction involving treatment assignment (*p* > 0.4) was significant, confirming that the plots allocated to the four treatments were comparable before treatment establishment.

#### 3.3.1. Soil Nitrite + Nitrate N Content in the First Investigated Period (2021–2022) in Case of Green Manure Application Compared with Fertilization and Control Treatments

**Figure 5:**
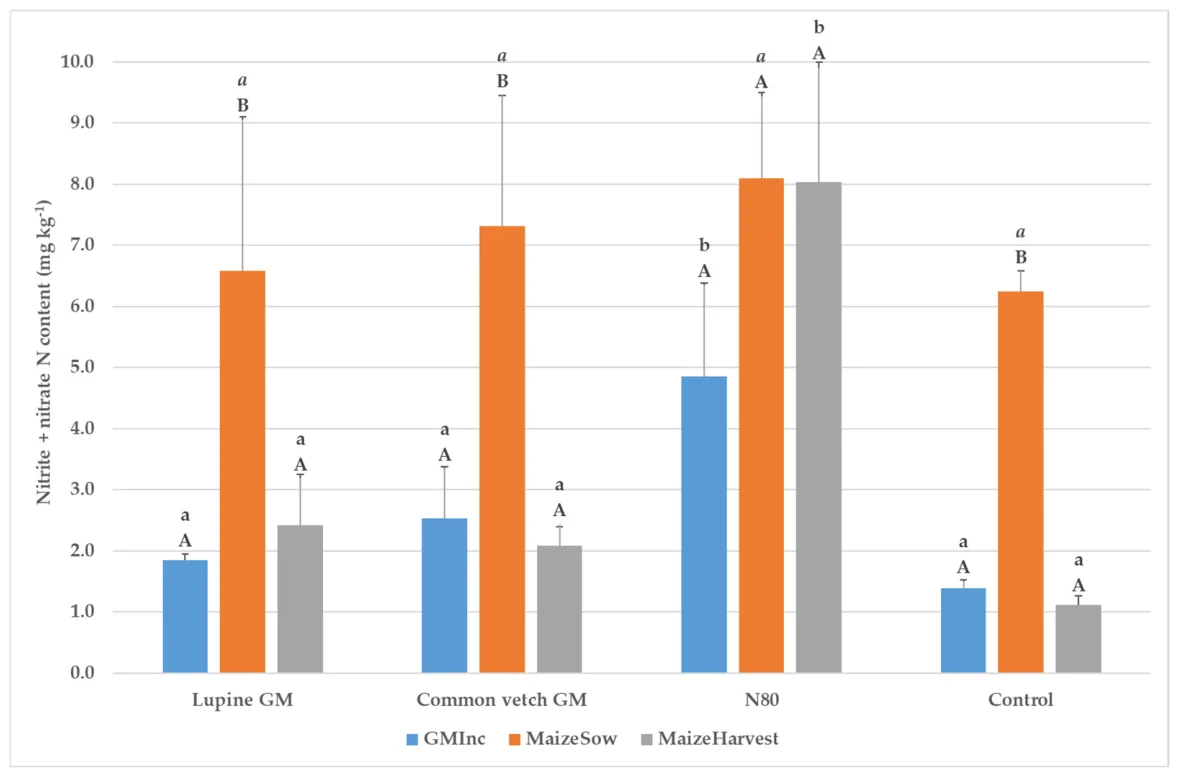
Impact of green manure application on the soil nitrite + nitrate N content at the 0–25 cm soil depth compared with fertilization and control treatments in 2021–2022. GM: Green manure. N80: Fertilization with 80 kg ha^−1^ N. GMInc: Measurement following the incorporation of the green manures. MaizeSow: Measurement following the sowing of maize. MaizeHarvest: Measurement following the harvest of maize. Note: Different lowercase letters indicate significant differences between treatments at the same sampling time, while different uppercase letters indicate significant differences between sampling times within the same treatment (Tukey’s test, *p* < 0.05).

Soil nitrite + nitrate N content was determined at three sampling times: following green manure incorporation (GMInc), following maize sowing (MaizeSow), and following maize harvest (MaizeHarvest), in each treatment at soil depths of 0–25 and 25–50 cm. In the initial year of the study (2021), limited precipitation during the green manure growing period coincided with the absence of statistically significant treatment effects in the 0–25 cm soil layer following green manure incorporation and maize sowing. Mineral N fertilizer was applied at the 4–6 leaf stage of maize. The mineral-fertilized treatment showed the highest nitrite + nitrate N content in the topsoil layer following maize harvesting (8.03 mg kg^−1^), which was nearly equivalent to the value recorded after maize sowing (8.03 mg kg^−1^). For the green manure treatments, the values measured after maize harvest (2.42 and 2.08 mg kg^−1^) were significantly lower than those measured after maize sowing (6.58 and 7.31 mg kg^−1^) in the 0–25 cm soil layer (Figure 5).

**Figure 6:**
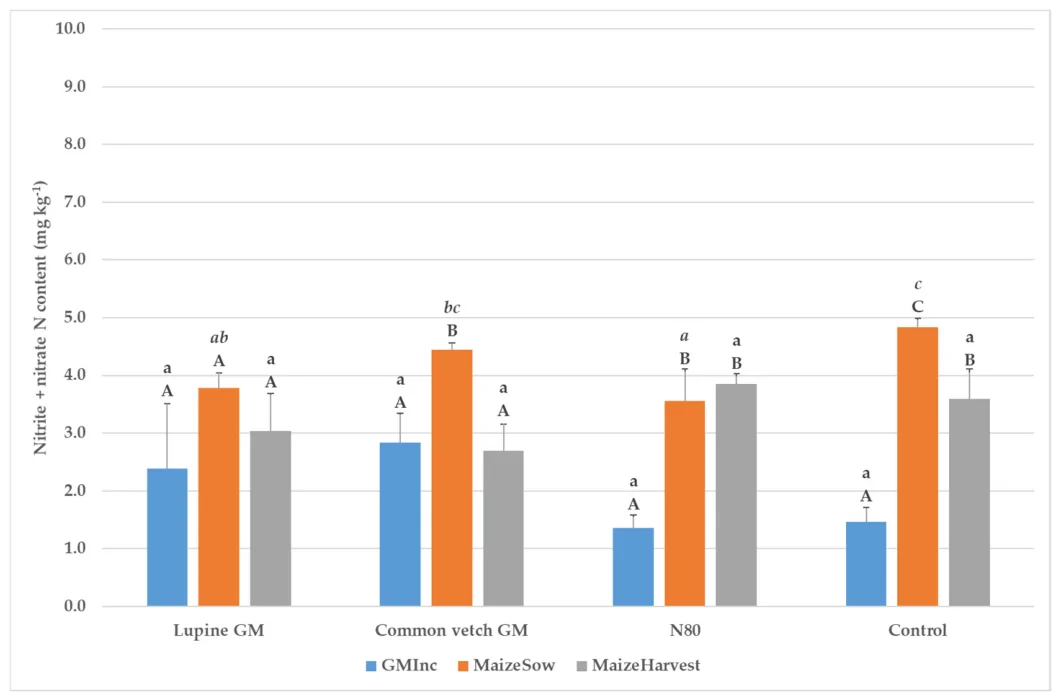
Impact of green manure application on the soil nitrite + nitrate N content at the 25–50 cm soil depth compared with fertilization and control treatments in 2021–2022. GM: Green manure. N80: Fertilization with 80 kg ha^−1^ N. GMInc: Measurement following the incorporation of the green manures. MaizeSow: Measurement following the sowing of maize. MaizeHarvest: Measurement following the harvest of maize. Note: Different lowercase letters indicate significant differences between treatments at the same sampling time, while different uppercase letters indicate significant differences between sampling times within the same treatment (Tukey’s test, *p* < 0.05).

No significant treatment effect was observed in the 25–50 cm soil layer after incorporation of the green manure plants. Following maize sowing, the soil nitrite and nitrate nitrogen content in areas treated with green manure plants exceeded the values measured for the mineral-fertilized treatment, with the highest value recorded in the control area. No significant differences between treatments were observed following maize harvest. However, the highest nitrogen content was also measured in the deeper soil layer of the mineral-fertilized treatment. In the case of fertilization, the nitrite and nitrate N values were higher following harvest than following sowing time (Figure 6).

#### 3.3.2. Soil Nitrite + Nitrate N Content in the Second Investigated Period (2022–2023) in Case of Green Manure Application Compared with Fertilization and Control Treatments

**Figure 7:**
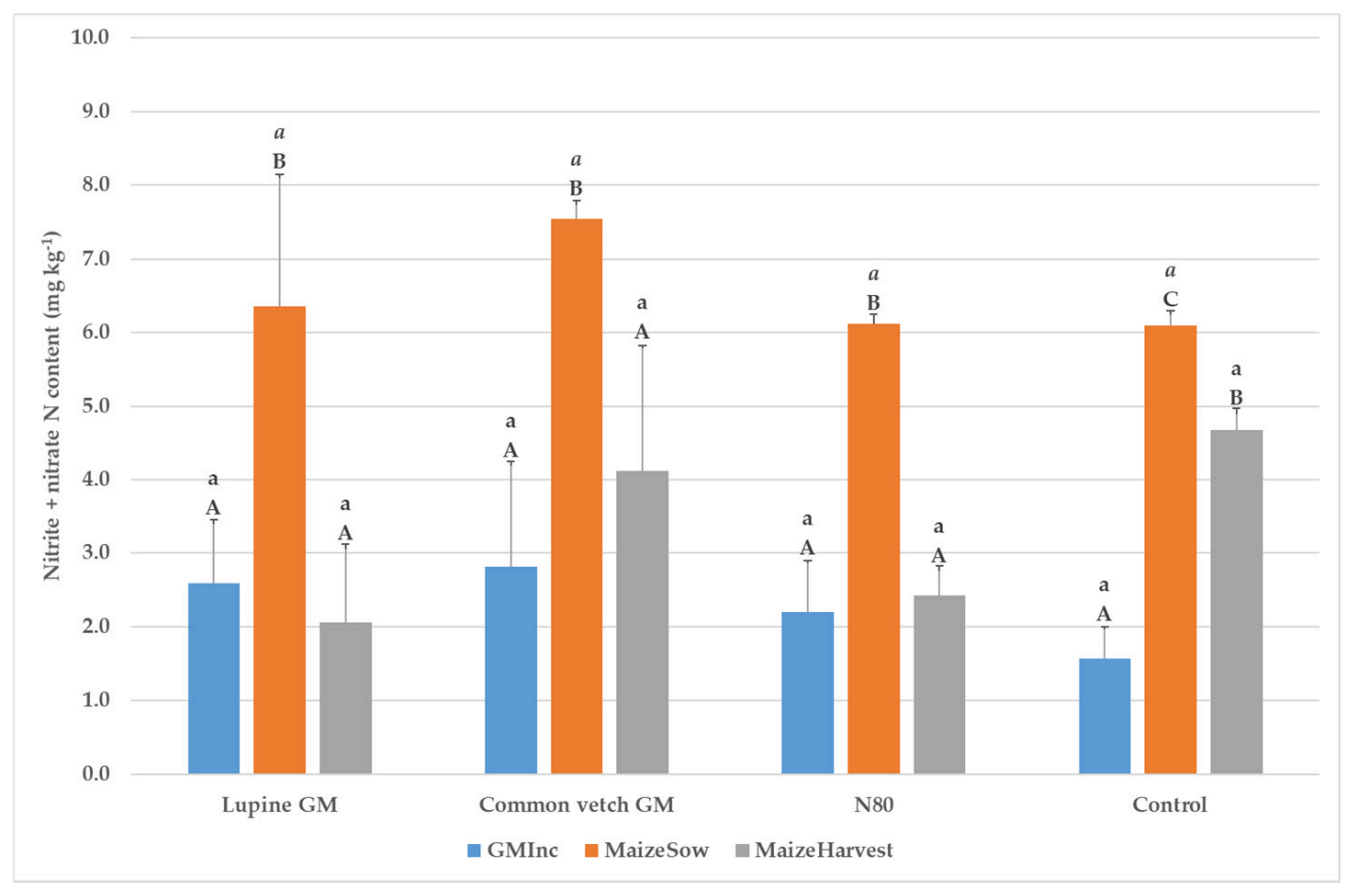
Impact of green manure application on the soil nitrite + nitrate N content at the 0–25 cm soil depth compared with fertilization and control treatments in 2022–2023. GM: Green manure. N80: Fertilization with 80 kg ha^−1^ N. GMInc: Measurement following the incorporation of the green manures. MaizeSow: Measurement following the sowing of maize. MaizeHarvest: Measurement following the harvest of maize. Note: Different lowercase letters indicate significant differences between treatments at the same sampling time, while different uppercase letters indicate significant differences between sampling times within the same treatment (Tukey’s test, *p* < 0.05).

In 2022, the green manure period received sufficient precipitation to allow optimal vegetative development of green manure crops. The highest nitrite + nitrate N content in the top 0–25 cm of the soil after incorporation of the green manure crops and after sowing of maize was measured for the green manured treatments, but the differences were not significant (Figure 7).

**Figure 8:**
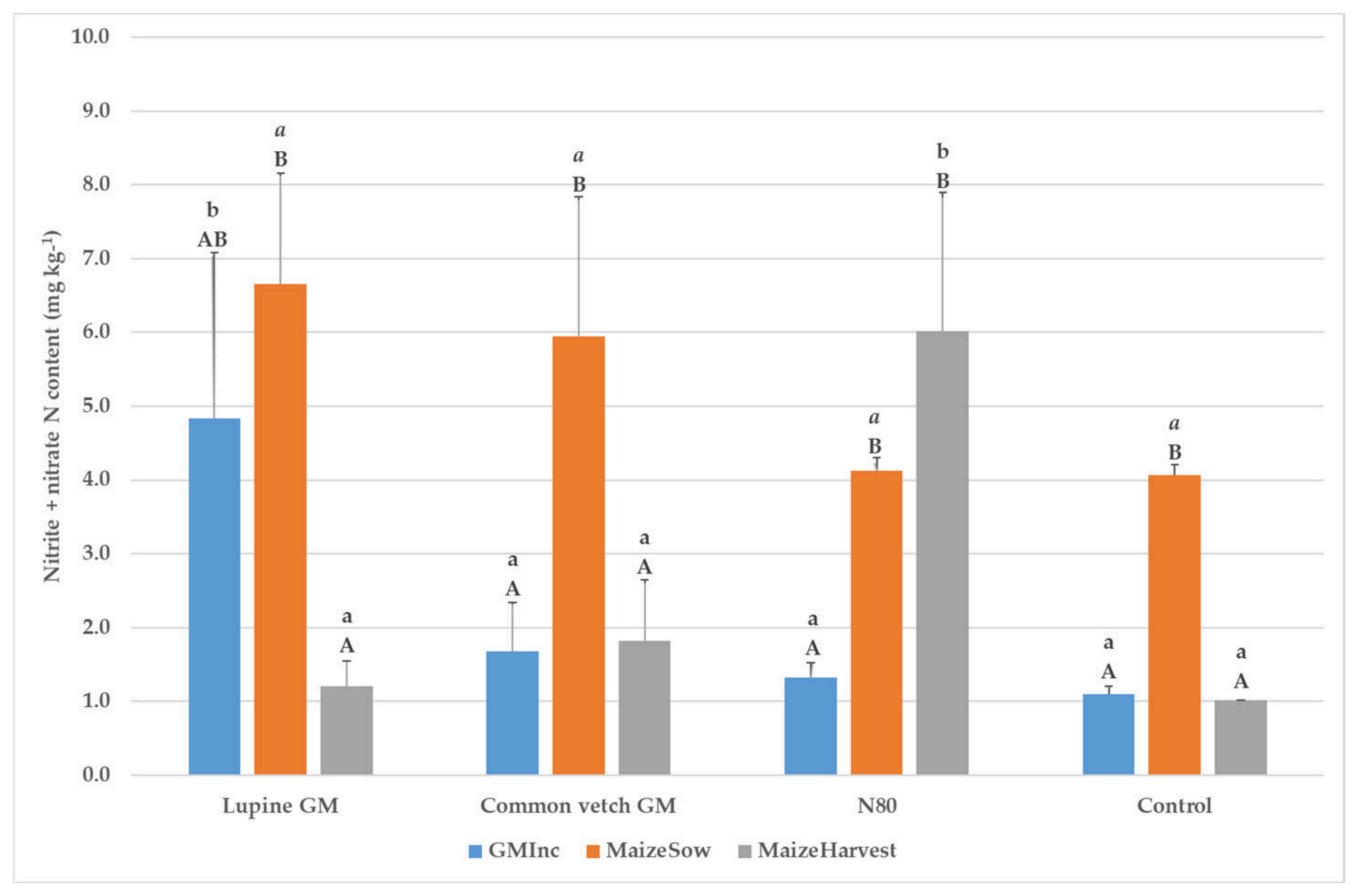
Impact of green manure application on the soil nitrite + nitrate N content at the 25–50 cm soil depth compared with fertilization and control treatments in 2022–2023. GM: Green manure. N80: Fertilization with 80 kg ha^−1^ N. GMInc: Measurement following the incorporation of the green manures. MaizeSow: Measurement following the sowing of maize. MaizeHarvest: Measurement following the harvest of maize. Note: Different lowercase letters indicate significant differences between treatments at the same sampling time, while different uppercase letters indicate significant differences between sampling times within the same treatment (Tukey’s test, *p* < 0.05).

Figure 8 illustrates that the nitrite and nitrate nitrogen content in the deeper soil layer (25–50 cm) after incorporation of the green manures was found to be significantly higher in the areas treated with lupine green manure (4.83 mg kg^−1^), while the measured value for the other treatments was less than 2 mg kg^−1^. Following maize sowing, the highest value was still observed in the area treated with lupine green manure. However, the effect of the treatment could not be statistically verified. Following maize harvest, the highest nitrite + nitrate N content was measured in the mineral-fertilized treatment (6.02 mg kg^−1^). In this case, the value measured after harvest was higher than that measured at the time of maize sowing (4.12 mg kg^−1^).

#### 3.3.3. Combined Effects of Growing Season, Nutrient Management Strategy and Sampling Date on Soil Nitrite + Nitrate N Content

To evaluate the combined effects of growing season, nutrient management strategy, and soil sampling date on soil nitrite + nitrate N content, repeated-measures ANOVA was performed separately for the 0–25 and 25–50 cm soil layers (Table 8). In both soil layers, nutrient management strategy significantly affected soil nitrite + nitrate N content, whereas the main effect of growing season was not significant. Soil sampling date had a highly significant effect at both depths (*p* < 0.001). In the 0–25 cm soil layer, the three-way interaction among growing season, nutrient management strategy and sampling date was significant (*p* = 0.010). In addition, the between-subject interaction between growing season and nutrient management strategy was also significant (*p* < 0.001). However, none of the within-subject two-way interactions involving sampling date reached statistical significance. In contrast, in the 25–50 cm soil layer, significant interactions were detected between sampling date and growing season (*p* = 0.004), sampling date and nutrient management strategy (*p* < 0.001), as well as among all three factors (*p* = 0.001), indicating that temporal changes in soil mineral N at the 25–50 cm layer depended on both growing season and nutrient management strategy.

#### 3.3.4. Pearson Correlation Between Precipitation and Soil Mineral Nitrogen Content Across Soil Depths

**Figure 9:**
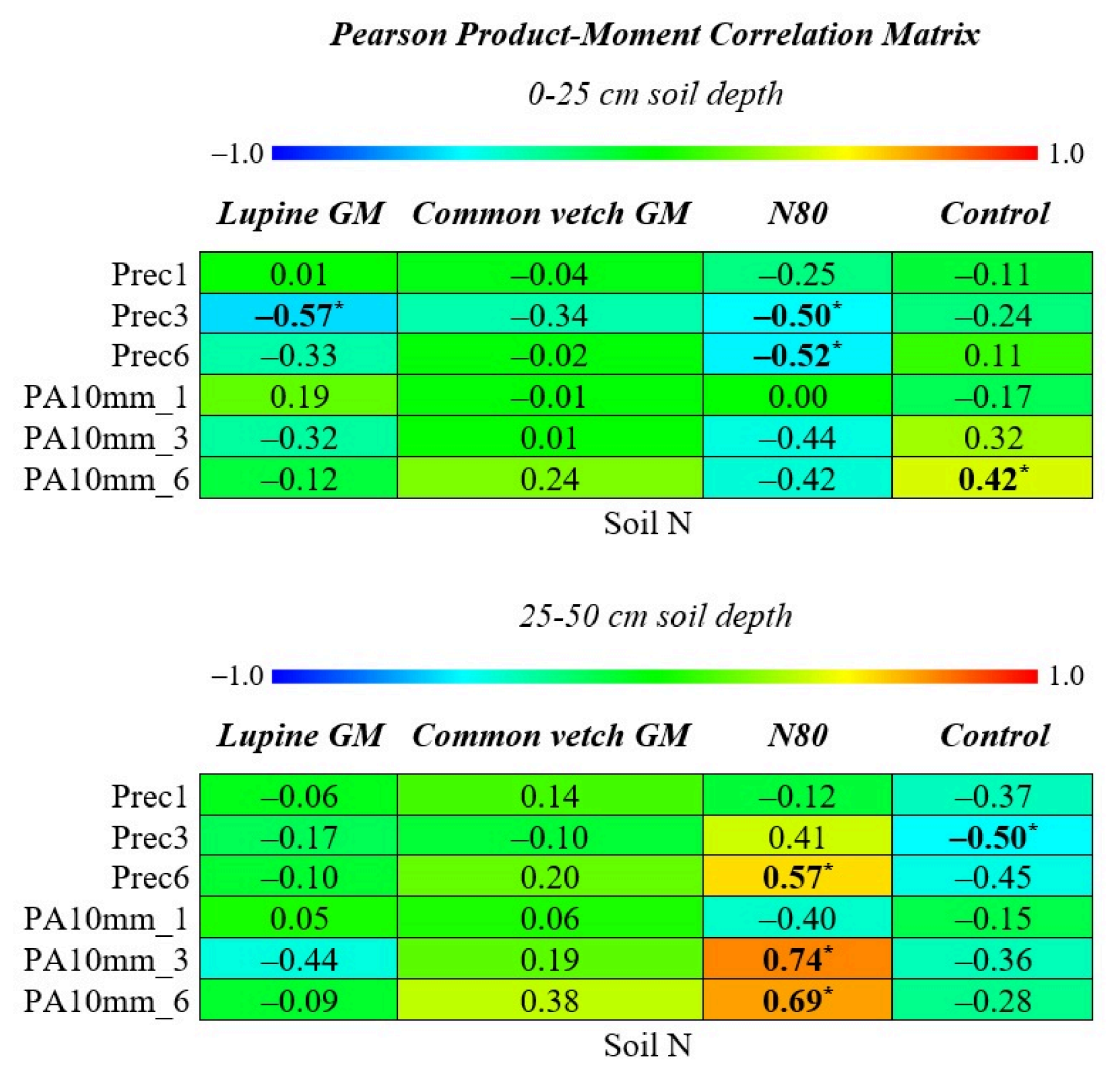
Pearson correlation coefficients between precipitation variables and soil mineral nitrogen (NO_2_^−^ + NO_3_^−^-N) content under different treatments and soil depths (0–25 cm and 25–50 cm). Prec1, Prec3, and Prec6 represent cumulative precipitation over 1, 3 and 6 months prior to sampling. PA10mm_1, PA10mm_3, PA10mm_6 represent the number of days with precipitation exceeding 10 mm over the same periods. Asterisks (*) indicate statistically significant correlations at *p* < 0.05.

The relationships between precipitation patterns and soil mineral nitrogen (NO_2_^−^ + NO_3_^−^-N) content were evaluated using Pearson correlation analysis across treatments and soil depths (Figure 9). In the 0–25 cm soil layer, precipitation variables showed predominantly negative correlations with soil mineral nitrogen content, particularly in the mineral-fertilized treatment. Significant negative relationships were observed for cumulative precipitation over three and six months (r = −0.50 and r = −0.52; *p* < 0.05, respectively), indicating that increased precipitation reduced nitrogen availability in the topsoil. A similar, albeit stronger, negative correlation was observed in the lupine treatment during the three-month precipitation period (r = −0.57, *p* < 0.05).

In contrast, correlations in the control treatment were weaker and less consistent, although a significant positive relationship was observed between the number of heavy rainfall events (>10 mm) over six months and soil nitrogen content (r = 0.42, *p* < 0.05), suggesting localized nitrogen accumulation in the absence of external inputs.

In the 25–50 cm soil layer, the pattern was reversed. In the mineral-fertilized treatment, strong positive correlations were observed between soil mineral nitrogen content and both cumulative precipitation (r = 0.57 for six months; *p* < 0.05) and rainfall intensity (r = 0.74 and r = 0.69 for 3- and 6-month periods, respectively; *p* < 0.05). These results indicate enhanced downward movement and accumulation of mineral nitrogen in the subsoil under increased precipitation conditions.

In contrast, green manure treatments (lupine and common vetch) showed weaker and less consistent relationships with precipitation variables in the deeper layer, suggesting more stable nitrogen retention and reduced susceptibility to leaching compared to mineral fertilization.

The results highlight a depth-dependent redistribution of nitrogen driven by precipitation, with depletion of mineral nitrogen in the topsoil and accumulation in deeper layers, particularly under mineral fertilization.

#### 3.3.5. Regression Analysis of Precipitation Effects on Soil Mineral Nitrogen Across Soil Depths

**Figure 10:**
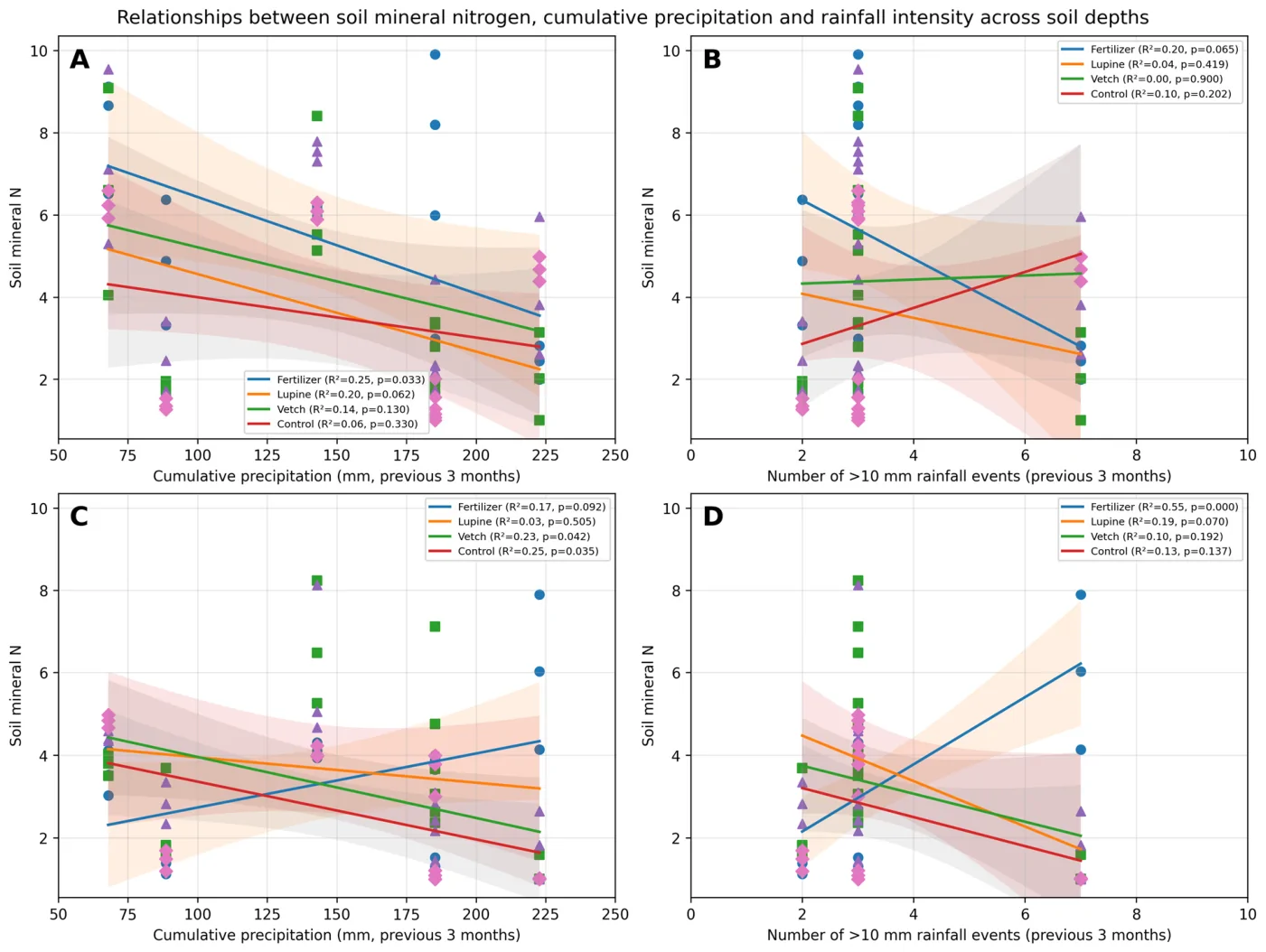
Relationships between soil mineral nitrogen (NO_2_^−^ + NO_3_^−^-N) and precipitation variables across soil depths. Panels (**A**,**C**) show relationships with cumulative precipitation (previous 3 months) in the 0–25 cm and 25–50 cm soil layers, respectively, while panels (**B**,**D**) present relationships with the number of precipitation events exceeding 10 mm over the same period in the 0–25 cm and 25–50 cm soil layers, respectively. Lines represent linear regressions for each treatment (lupine, common vetch, fertilizer and control), with shaded areas indicating 95% confidence intervals. R^2^ and *p*-values are shown for each regression. Individual data points are shown as circles (mineral N fertilizer), triangles (white lupine GM), squares (common vetch GM), and diamonds (control).

Regression analysis further clarified the relationships between precipitation patterns and soil mineral nitrogen (NO_2_^−^ + NO_3_^−^-N) content across treatments and soil depths (Figure 10).

In the 0–25 cm soil layer, cumulative precipitation showed a consistent negative relationship with soil mineral nitrogen content across all treatments (Figure 10A). This trend was most pronounced in the mineral-fertilized treatment (R^2^ = 0.25, *p* = 0.033), indicating that increasing precipitation reduced nitrogen availability in the topsoil. Similar negative trends were observed for green manure treatments, although these relationships were weaker and not statistically significant.

When precipitation intensity was considered (Figure 10B), contrasting responses were observed among treatments. In mineral-fertilized plots, soil nitrogen content decreased with increasing frequency of heavy rainfall events, while in the control treatment a positive trend was detected, suggesting differences in nitrogen retention and redistribution dynamics under varying input conditions.

In the 25–50 cm soil layer, the relationships differed markedly (Figure 10C,D). In contrast to the topsoil, cumulative precipitation increased soil mineral nitrogen content in the subsoil of mineral-fertilized plots. This effect was even more pronounced when rainfall intensity was considered (Figure 10D), where a strong positive relationship was observed in the mineral-fertilized treatment (R^2^ = 0.55, *p* < 0.001).

These results clearly indicate a depth-dependent redistribution of nitrogen, in which increased precipitation leads to depletion of mineral nitrogen in the topsoil and its accumulation in deeper soil layers. This effect was most pronounced in the mineral-fertilized treatment, suggesting greater mobility and leaching susceptibility of mineral nitrogen than of nitrogen derived from green manure.

### 3.4. Principal Component Analysis of Soil Nitrogen Dynamics, Precipitation and Crop Performance

Principal component analysis (PCA) was performed to explore the multivariate relationships between soil mineral nitrogen (NO_2_^−^ + NO_3_^−^-N), precipitation variables, and crop performance indicators across treatments and soil depths.

In the 0–25 cm soil layer, the first principal component (PC1) explained 61.6% of the total variance, indicating a dominant gradient along which crop performance variables, including maize yield, plant height and green manure biomass, were aligned. These variables showed an opposing orientation relative to precipitation variables, particularly cumulative precipitation over longer periods (3 and 6 months), suggesting that increased rainfall may be associated with reduced nitrogen availability and crop performance in the topsoil.

**Figure 11:**
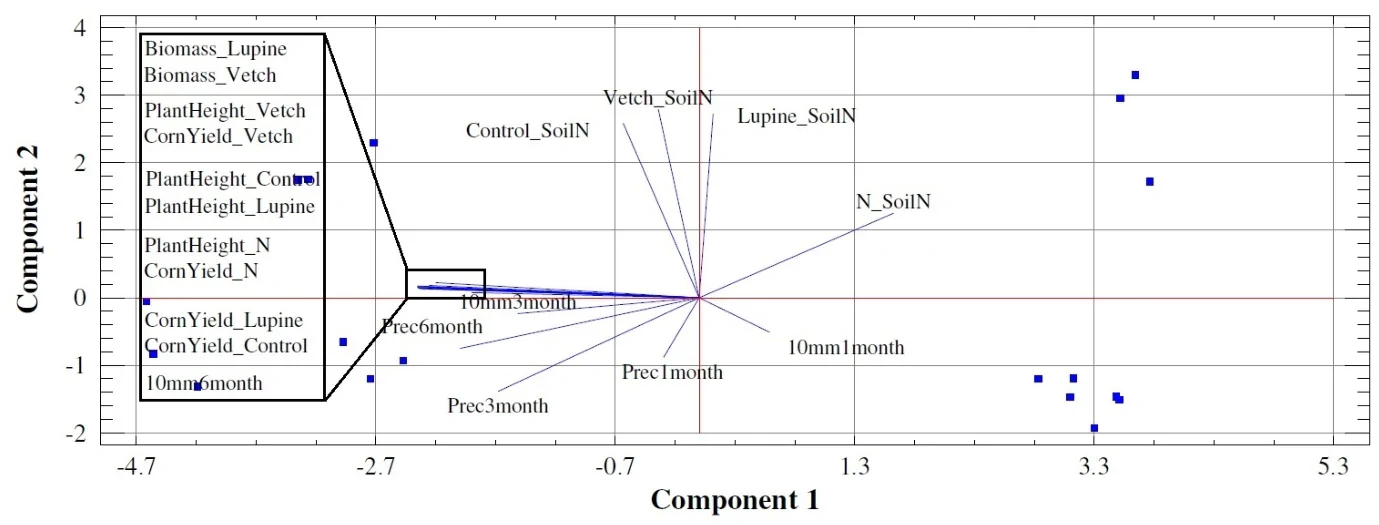
Principal component analysis (PCA) biplot showing the relationships between soil mineral nitrogen, precipitation variables and crop performance indicators in the 0–25 cm soil layer. Blue arrows represent explanatory variables (soil mineral nitrogen and precipitation indices), while black labels indicate treatment-specific yield, biomass, and plant height variables. The direction and length of vectors indicate the strength and nature of the relationships among variables. CornYield_Control, CornYield_N, CornYield_Lupine, and CornYield_Vetch represent maize grain yield under the control, mineral nitrogen fertilization, lupine green manure, and common vetch green manure treatments, respectively. PlantHeight_Control, PlantHeight_N, PlantHeight_Lupine, and PlantHeight_Vetch denote maize plant height, while Biomass_Lupine and Biomass_Vetch indicate the biomass production of the respective green manure crops. Control_SoilN, N_SoilN, Lupine_SoilN, and Vetch_SoilN represent soil mineral nitrogen (NO_2_^−^ + NO_3_^−^) under the respective treatments. Prec1month, Prec3month, and Prec6month indicate cumulative precipitation during the previous 1, 3, and 6 months, respectively, while 10mm1month, 10mm3month, and 10mm6month represent the number of precipitation events ≥10 mm during the previous 1, 3, and 6 months.

The second principal component (PC2) was primarily associated with soil mineral nitrogen content across treatments, indicating that nitrogen dynamics in the topsoil were influenced by multiple interacting factors rather than clearly separated treatment effects. Mineral fertilizer-derived nitrogen (N_SoilN) showed a partially opposing orientation relative to precipitation variables, indicating a weak or inverse association in the topsoil (Figure 11).

**Figure 12:**
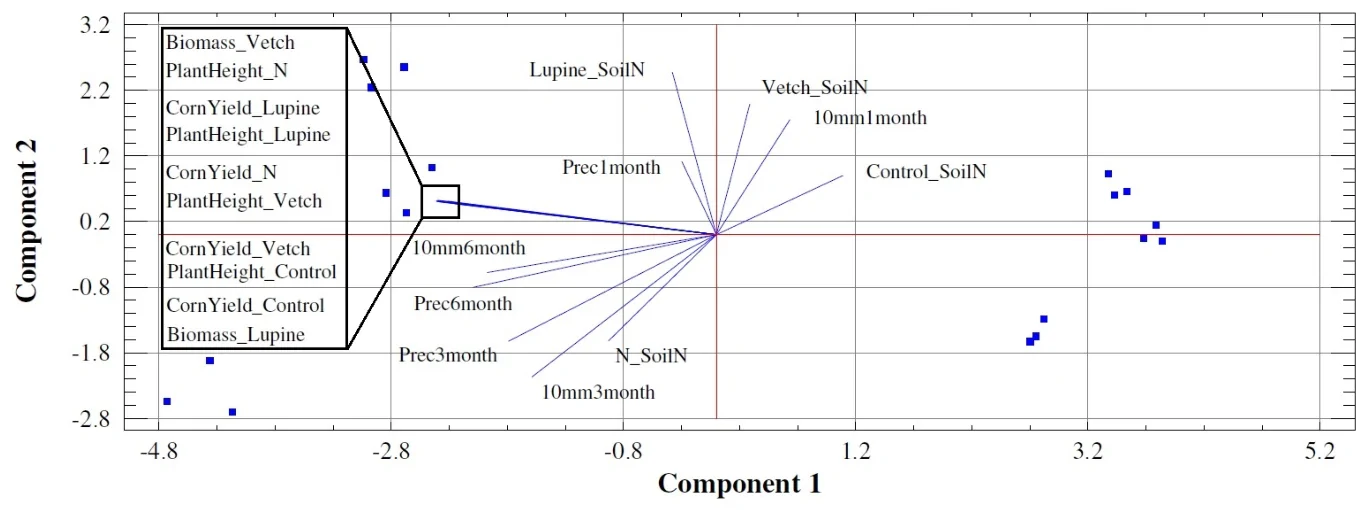
Principal component analysis (PCA) biplot showing the relationships between soil mineral nitrogen, precipitation variables and crop performance indicators in the 25–50 cm soil layer, illustrating the stronger association between mineral fertilizer-derived nitrogen and precipitation variables in the subsoil. Blue arrows represent explanatory variables (soil mineral nitrogen and precipitation indices), while black labels indicate treatment-specific yield, biomass, and plant height variables. The direction and length of vectors indicate the strength and nature of the relationships among variables. CornYield_Control, CornYield_N, CornYield_Lupine, and CornYield_Vetch represent maize grain yield under the control, mineral nitrogen fertilization, lupine green manure, and common vetch green manure treatments, respectively. PlantHeight_Control, PlantHeight_N, PlantHeight_Lupine, and PlantHeight_Vetch denote maize plant height, while Biomass_Lupine and Biomass_Vetch indicate the biomass production of the respective green manure crops. Control_SoilN, N_SoilN, Lupine_SoilN, and Vetch_SoilN represent soil mineral nitrogen (NO_2_^−^ + NO_3_^−^) under the respective treatments. Prec1month, Prec3month, and Prec6month indicate cumulative precipitation during the previous 1, 3, and 6 months, respectively, while 10mm1month, 10mm3month, and 10mm6month represent the number of precipitation events ≥10 mm during the previous 1, 3, and 6 months.

In the 25–50 cm soil layer, a similar overall variance structure was observed, with PC1 explaining 60.7% of the total variance (Figure 12). Mineral nitrogen associated with fertilization (N_SoilN) showed a distinct separation from nitrogen forms derived from green manure treatments. N_SoilN was closely aligned with precipitation variables, indicating a strong dependence on rainfall-driven processes and enhanced mobility within the soil profile.

In contrast, soil nitrogen associated with lupine and common vetch was less strongly associated with precipitation gradients and remained more clustered, suggesting a more stable nitrogen pool with reduced susceptibility to downward movement.

This separation indicates that mineral fertilizer-derived nitrogen is more prone to precipitation-driven redistribution, while nitrogen originating from green manure is retained more effectively within the soil system.

## 4. Discussion

Following the harvest of the cash crops, we hypothesized that leguminous green manure crops (lupine and common vetch) would enhance nutrient availability for the subsequent crop through both the incorporation of biomass and the contribution of biologically fixed nitrogen. This dual effect was expected to improve soil conditions by increasing organic matter content and providing a more sustained nitrogen supply [3,4,45].

### 4.1. Impact of Precipitation on the Above-Ground Biomass Weight of Leguminous Green Manures

Previous studies have reported a positive relationship between growing-season precipitation and the above-ground biomass production of common vetch, and similar responses have been observed for lupine [46,47]. In the present study, the above-ground biomass production of the green manure crops was associated with precipitation during August and September. In 2021, precipitation during this period was significantly lower than in 2022. The biomass yield of the tested leguminous green manure crops showed significant differences across the years studied (Table 5). In addition to rainfall, differences in the performance of the preceding cash crops may also have contributed to the variation in green manure biomass production. While 2021 was favorable for cash crop development, the severe drought in 2022 resulted in substantially lower yields. Consequently, more favorable conditions for green manure establishment, such as reduced competition for residual soil water and nutrients, may have occurred, although these factors were not directly measured in the present study.

### 4.2. Impact of Leguminous Green Manures and N Fertilization on Maize Yield

The input of nutrients such as nitrogen (N) and other minerals through the incorporation of leguminous green manure might directly affect crop yield [48,49]. In our experiment, incorporating leguminous green manure crops provided a substantial nitrogen input that significantly influenced maize yield. Across the years studied, lupine and common vetch treatments achieved yields comparable to or exceeding those obtained with 80 kg ha^−1^ of mineral nitrogen fertilization.

The observed yield response is consistent with the biological nitrogen fixation capacity of legumes and the gradual release of nitrogen during residue mineralization reported in previous studies, which may improve synchronization between nitrogen availability and crop demand. Estimated nitrogen replacement values reported in the literature (58–184 kg N ha^−1^) [20,50] are consistent with our findings. These findings are consistent with previous studies reporting that green manure application in crop rotations significantly reduces fertilizer nitrogen requirements by 25–50% without yield losses. Green manure achieves superior nitrogen use efficiency (79% apparent recovery versus 63% for mineral fertilizer) and supports equivalent or greater crop yields [51,52]. Previous studies have also reported that green manure incorporation improved dissolved organic nitrogen and mineral nitrogen status at both early and mid-late stages of maize growth, with positive indirect effects on dry matter production through enhanced nitrogen uptake [53].

Importantly, under drought conditions (2022), green manure treatments significantly outperformed mineral fertilization. This may suggest a buffering effect of organic amendments, likely associated with improved soil structure, greater water retention and a more gradual nitrogen supply reported for leguminous green manures. In contrast, mineral-fertilized plots showed yield depression, which may indicate that mineral nitrogen may exacerbate stress under water-limited conditions.

### 4.3. Impact of Leguminous Green Manures and N Fertilization on the Nitrite and Nitrate N Content of Soil

The repeated-measures ANOVA indicated that soil sampling date and nutrient management strategy were the primary factors influencing soil mineral nitrogen dynamics, whereas the main effect of growing season was not significant. Several significant interactions, particularly in the 25–50 cm soil layer, indicated that the temporal response of soil mineral nitrogen depended on both nutrient management strategy and growing season (Table 8).

When the growing season of the green manure crops received sufficient rainfall to produce an optimal amount of biomass (2022), the nitrite + nitrate N content of the soil in May of the following year exceeded the nitrite + nitrate N content of the mineral-fertilized and control treatments. These observations are consistent with recommendations that green manure crops should be sown as early as possible after cereal harvest to maximize biomass production and nutrient accumulation before spring-sown crops [54,55].

In 2022, the highest N content in the 0–25 cm soil layer following maize harvest was measured in the mineral-fertilized treatment, and it was almost identical to the value measured after maize sowing (Figure 5). The persistence of high mineral N concentrations in the mineral-fertilized treatment after maize harvest suggests that maize nitrogen uptake was limited under the severe drought conditions of 2022. In soils treated with green manure crops, the values measured after maize harvest were lower than those measured after maize sowing in both the 0–25 cm and 25–50 cm soil layers (Figure 5 and Figure 6). The lower post-harvest mineral N concentrations observed in the green manure treatments are consistent with more effective nitrogen utilization by maize, as supported by the higher grain yields (Table 7). The temporal pattern observed in the present study also agrees with the conceptual framework described by Li et al. [56], who identified temporal dynamics of N release, soil texture, biomass composition and fertilizer interactions as the principal factors governing soil nitrate and nitrite availability after green manure incorporation. In sandy soils, these processes are particularly important because nitrogen mobility is strongly influenced by rainfall and drainage conditions. Legume green manures were found to significantly increase total soil nitrogen and the cash crop following green manure treatments had higher nitrogen uptake and yields than those receiving synthetic fertilizers alone [57].

In a controlled study, the incorporation of lupine significantly increased nitrogen uptake in maize aboveground biomass compared to bare soil. This effect was observed across both sandy and clay soils, indicating the broad applicability of lupine as a nitrogen-enhancing cover crop [58]. In our study, the highest nitrite + nitrate N content in the 25–50 cm soil layer after incorporating green manure crops in the year 2022–2023, with good rainfall, was measured in areas treated with lupine green manure. After maize sowing, the highest value was still observed in the area treated with lupine green manure, but the treatment effect was not statistically significant. However, after maize harvest, the highest nitrite + nitrate N content was observed in the mineral-fertilized treatment. In this case, the value measured after harvest was higher than the value measured at the time of maize sowing. The higher mineral N concentrations measured in the 25–50 cm layer after maize harvest are consistent with downward movement of fertilizer-derived nitrogen during the wetter growing season (Figure 10), although nitrogen transport was not directly measured in the present study. The stronger and more numerous significant interactions detected by the repeated-measures ANOVA in the 25–50 cm soil layer further support the interpretation that nitrogen dynamics were more responsive to temporal changes in environmental conditions in the deeper soil profile than in the topsoil. A similar finding was reported by Salmerón [48], who found that green manure crops reduced N leaching risks, as soil N content in spring and at maize harvest was lower than in the mineral-fertilized control treatment. Similar results were reported by Fowler et al. [59], who observed substantially lower cumulative N leaching losses in lupine-based systems (4.1–4.9 kg N ha^−1^) compared with bare fallow treatments (8.4 kg N ha^−1^). Likewise, Willumsen and Thorup-Kristensen [60] demonstrated that leguminous green manures, particularly hairy vetch, were the most effective legume green manures for increasing mineral N availability for subsequent crops and reducing leachable N by 50–85%. Regarding Figure 11, the depression of maize yield in mineral-fertilized plots during the extreme drought of 2022 (0.90 t ha^−1^ vs. 1.41 t ha^−1^ in the control) suggests a negative interaction between nitrogen mineral application and water scarcity and suggests that mineral nitrogen fertilization was less effective under severe water limitation than the nitrogen supplied through leguminous green manures. In contrast, the superior performance of the green manure treatments under drought conditions may reflect several mechanisms reported in the literature, including gradual nitrogen release and improved soil physical properties; however, these mechanisms were not directly investigated in the present study.

Besides biological N_2_ fixation and residue mineralization, white lupine may also influence soil inorganic nitrogen dynamics through species-specific rhizosphere processes. Under phosphorus-deficient conditions, white lupine develops cluster (proteoid) roots that can induce localized rhizosphere acidification through proton release, potentially affecting nutrient availability and microbial activity [61,62,63]. However, current evidence does not demonstrate a direct causal relationship between root-induced rhizosphere acidification and soil inorganic nitrogen dynamics, and the magnitude of this effect appears to depend strongly on soil conditions [64,65,66]. Because cluster-root abundance, rhizosphere pH and associated microbial processes were not quantified in the present study, the contribution of this mechanism cannot be distinguished from other processes such as plant N uptake, microbial immobilization or residue mineralization.

The baseline soil mineral N analysis also revealed a significant effect of crop rotation prior to treatment establishment. As soil sampling was performed before the application of green manure and mineral fertilization treatments, this difference most likely reflects the different preceding cereal crops in the two staggered crop rotations (triticale in Crop Rotation I and oat in Crop Rotation II), rather than treatment effects. Importantly, no significant effect of future treatment assignment was detected at baseline, confirming that the plots allocated to the four treatments were comparable before treatment establishment.

### 4.4. Role of Precipitation in Nitrogen Redistribution Across Soil Depths

The combined results of the correlation analysis, regression modeling, and principal component analysis consistently indicated a strong association between precipitation and the distribution of soil mineral nitrogen within the soil profile. Similar relationships between precipitation and nitrate transport have been reported in coarse-textured soils, where precipitation shortly after fertilizer application was identified as one of the primary drivers of nitrogen loss through leaching [67]. According to the results of a study [68], rapid nitrification following urea application, combined with intensive rainfall, accelerated nitrate leaching into deeper layers, resulting in fertilizer losses. Moreover, future climate projections indicate that replacing synthetic fertilizers with green manure may help reduce peak N losses under future irrigation conditions [69].

Pearson correlation analysis revealed a consistent negative relationship between precipitation and soil mineral nitrogen content in the 0–25 cm layer, particularly in mineral-fertilized treatments. In contrast, strong positive correlations were observed in the 25–50 cm layer, consistent with greater nitrogen accumulation in deeper soil horizons under increased precipitation.

Regression analysis showed the same overall trend, with higher cumulative precipitation and rainfall intensity associated with reduced nitrogen availability in the topsoil and increased nitrogen content in the subsoil. This effect was most pronounced in the mineral-fertilized treatment, where rainfall intensity showed a strong positive relationship with subsoil nitrogen content.

Principal component analysis revealed a clear separation between nitrogen derived from mineral fertilization and nitrogen associated with green manure treatments. In the subsoil layer (25–50 cm), mineral nitrogen was closely aligned with precipitation variables, suggesting stronger associations with rainfall-driven processes and increased mobility within the soil profile. In contrast, nitrogen associated with green manure treatments showed weaker alignment with precipitation gradients and remained more clustered, which may reflect a more stable nitrogen pool with reduced susceptibility to leaching and a more gradual release pattern. The weaker association between precipitation variables and green manure-derived nitrogen observed in the PCA may indicate a slower and more buffered release pattern. This interpretation is supported by Regina et al. [70], who reported that residual N from clover biomass mulched in autumn or spring did not increase leaching losses during the growing season or the year following harvest.

Together, these results suggest a depth-dependent redistribution of soil mineral nitrogen associated with precipitation. Compared with the green manure treatments, the mineral-fertilized treatment showed stronger associations between precipitation and subsoil mineral nitrogen, consistent with greater downward movement of mineral nitrogen under wetter conditions. Although the statistical analyses consistently identified strong relationships between precipitation and soil mineral nitrogen distribution, the underlying transport and mineralization processes were not directly measured, and therefore the observed relationships should not be interpreted as direct evidence of causal mechanisms.

The present manuscript reports the results of two maize growing seasons of an ongoing long-term field experiment and should therefore be interpreted as a two-year pilot study. Although the contrasting weather conditions provided an opportunity to evaluate treatment responses under markedly different production environments, the current dataset does not allow the independent effects of individual climatic variables to be fully separated from crop rotation effects. Data from subsequent years of the experiment are currently being processed and will enable a more robust assessment of long-term treatment effects, climatic drivers and growing season × nutrient management strategy interactions in future publications.

## 5. Conclusions

This two-year pilot study demonstrated that leguminous green manure crops (white lupine and common vetch) can provide agronomic performance comparable to mineral nitrogen fertilization under contrasting precipitation conditions in maize production systems.

The combined analysis showed strong associations between precipitation patterns and soil mineral nitrogen distribution. Increased precipitation was associated with lower mineral nitrogen content in the topsoil (0–25 cm) and higher mineral nitrogen content in the subsoil (25–50 cm), suggesting increased downward redistribution within the soil profile.

These associations were particularly pronounced in the mineral-fertilized treatment, where soil mineral nitrogen showed stronger relationships with precipitation variables than in the green manure treatments. In contrast, soil mineral nitrogen in the green manure treatments exhibited weaker associations with precipitation, consistent with a more gradual pattern of nitrogen release reported in previous studies.

Under drought conditions, green manure treatments maintained substantially higher maize grain yields than mineral nitrogen fertilization, highlighting their potential to improve yield stability in water-limited environments.

The results indicate that integrating leguminous green manure crops into crop rotations may contribute to more stable soil nitrogen dynamics and reduced reliance on mineral nitrogen fertilization in sandy soils under variable precipitation conditions.

Although the observed relationships are consistent with mechanisms reported in the literature, the present study represents two years of an ongoing long-term experiment. Therefore, further multi-year evaluations, together with direct measurements of nitrogen transformation and transport processes, are required to better distinguish the effects of climatic variability from crop rotation and to clarify the mechanisms governing soil mineral nitrogen dynamics.

## Figures and Tables

**Table 1:** General soil chemical properties of Crop Rotations I and II determined in August 2021 at the beginning of the evaluation period.

Soil Property	CR1 0–25 cm (Mean ± SD)	CR2 0–25 cm (Mean ± SD)	CR1 25–50 cm (Mean ± SD)	CR2 25–50 cm (Mean ± SD)	Crop Rotation *p*-Value	Soil Depth *p*-Value	Rotation × Depth *p*-Value
pH (KCl, 1:2.5)	7.30 ± 0.49	7.35 ± 0.41	7.35 ± 0.50	7.43 ± 0.35	0.657	0.698	0.918
Carbonated lime (m/m%)	1.35 ± 1.90	1.38 ± 1.41	2.09 ± 2.32	2.33 ± 2.57	0.850	0.247	0.904
Organic matter (m/m%)	1.58 ± 0.19	1.51 ± 0.20	1.36 ± 0.21	1.34 ± 0.23	0.604	**0.009**	0.863
NO_2_^−^ + NO_3_^−^-N (mg kg^−1^ d. m.)	3.17 ± 2.19	2.16 ± 0.64	2.32 ± 0.88	2.84 ± 1.71	0.627	0.446	0.835
Available K_2_O (mg kg^−1^ d. m.)	263.64 ± 74.16	274.36 ± 66.83	203.04 ± 93.97	199.21 ± 73.98	0.903	**0.020**	0.791
Available P_2_O_5_ (mg kg^−1^ d.m.)	261.21 ± 100.92	262.92 ± 78.97	189.61 ± 119.92	173.39 ± 74.58	0.828	**0.024**	0.792

Values are presented as mean ± SD (n = 8). *p*-values were obtained by two-way ANOVA with crop rotation (CR1 vs. CR2) and soil depth (0–25 cm vs. 25–50 cm) as fixed factors. Abbreviations: CR1—Crop Rotation 1 CR2—Crop Rotation 2; m/m%—mass percentage; d. m.—dry matter. Bold values indicate statistical significance at *p* < 0.05.

**Table 2:** Crop sequence of the examined crop rotations until maize harvest in 2023.

Year/Period	CR1	CR2
2020—CCP	Oat	Triticale
2020—GMP	Green manures	Green manures
2021—CCP	Triticale	Oat
2021—GMP	Green manures	Green manures
2022—CCP	Maize	Triticale
2022—GMP	Fallow	Green manures
2023—CCP	Triticale	Maize

CR: Crop Rotation; CCP: Cash Crop Period referring to the growing period of main crops. GMP: Green Manure Period referring to the vegetation period of the green manures.

**Table 3:** Baseline soil mineral nitrogen (NO_3_^−^-N + NO_2_^−^-N) content of the experimental plots before the first green manure (GM) sowing (10 August 2020) in Crop Rotations I and II at the 0–25 cm and 25–50 cm soil depth.

Treatment Assignment	Crop Rotation I. (Preceding Crop: Triticale)		Crop Rotation II. (Preceding Crop: Oat)	
	0–25 cm (mg kg^−1^ d. m.)	25–50 cm (mg kg^−1^ d. m.)	0–25 cm (mg kg^−1^ d. m.)	25–50 cm (mg kg^−1^ d. m.)
Lupine GM	2.16 ± 0.42	1.17 ± 0.17	2.96 ± 0.43	2.57 ± 0.90
Common vetch GM	2.76 ± 0.53	1.37 ± 0.17	2.87 ± 0.47	2.13 ± 0.48
Mineral N (N80)	2.05 ± 0.80	1.24 ± 0.22	3.06 ± 0.33	2.16 ± 0.32
Control	2.54 ± 0.41	1.12 ± 0.12	2.91 ± 0.17	2.16 ± 0.19

Values are presented as mean ± SD (n = 3), based on the three replicate plots that were consistently sampled throughout the experiment. Soil samples were collected before the establishment of the green manure and fertilization treatments. Baseline mineral N data were further evaluated using factorial repeated-measures ANOVA with crop rotation and future treatment assignment as between-subject factors and soil depth as the within-subject factor; the corresponding statistical results are presented in Section 3.

**Table 4:** Timeline of field operations and measurements.

Operation/Year	2021/2022	2022/2023
GM sowing	12 August	3 August
GM sampling	27 October	26 October
GM termination	28 October	27 October
Soil sampling	8 November	2 November
Maize sowing	3 May	3 May
Soil sampling	16 May	8 May
Fertilization (80 kg ha^−1^ N) *	1 June	15 June
Maize harvest	14 October	5 October
Soil sampling	2 November	12 November

* only in case of the mineral-fertilized treatments. GM: Green Manures (common vetch and lupine).

**Table 5:** Above-ground fresh biomass of white lupine (*Lupinus albus* L.) and common vetch (*Vicia sativa* L.) in 2021 and 2022, and results of a two-way ANOVA.

Growing Season		Legume Species	Above-Ground Fresh Biomass (t ha^−1^; Mean ± SD)
2021		White lupine (*Lupinus albus* L.)	0.41 ± 0.30
		Common vetch (*Vicia sativa* L.)	0.73 ± 0.11
2022		White lupine (*Lupinus albus* L.)	3.04 ± 0.66
		Common vetch (*Vicia sativa* L.)	2.97 ± 0.46
**Source of variation**	df	F	*p*-value
Growing season	1	126.996	<0.001
Legume species	1	0.347	0.567
Growing season × Legume species	1	0.811	0.386

Values are presented as mean ± standard deviation (SD) of four independent field replicates. F- and *p*-values correspond to the main effects of growing season and legume species, and their interaction, obtained from a two-way ANOVA.

**Table 6:** Effect of nutrient management strategy on maize plant height in 2022 and 2023, and results of a two-way ANOVA.

Growing Season	Nutrient Management Strategy		Plant Height (cm; Mean ± SD)
2022	White lupine GM		180.86 ± 3.60
	Common vetch GM		184.25 ± 6.36
	Mineral N (N80)		149.17 ± 24.67
	Control		151.83 ± 10.49
2023	White lupine GM		304.00 ± 1.42
	Common vetch GM		307.44 ± 1.00
	Mineral N (N80)		305.25 ± 7.17
	Control		304.94 ± 5.27
**Source of variation**	df	F	*p*-value
Growing season	1	1444.915	<0.001
Nutrient management strategy	3	6.830	0.002
Growing season × Nutrient management strategy	3	6.195	0.003

Values are presented as mean ± standard deviation (SD) of four independent field replicates. F- and *p*-values correspond to the main effects of growing season and nutrient management strategy, and their interaction, as determined by two-way ANOVA.

**Table 7:** Effect of nutrient management strategy on maize grain yield in 2022 and 2023, and results of a two-way ANOVA.

Growing Season	Nutrient Management Strategy		Yield (t ha^−1^; Mean ± SD)
2022	White lupine GM		4.21 ± 0.23
	Common vetch GM		4.11 ± 0.27
	Mineral N (N80)		0.90 ± 0.40
	Control		1.61 ± 0.57
2023	White lupine GM		10.87 ± 0.43
	Common vetch GM		11.68 ± 0.65
	Mineral N (N80)		11.71 ± 1.45
	Control		9.45 ± 1.07
**Source of variation**	df	F	*p*-value
Growing season	1	967.557	<0.001
Nutrient management strategy	3	17.205	<0.001
Growing season × Nutrient management strategy	3	11.624	<0.001

Values are presented as mean ± standard deviation (SD) of four independent field replicates. F- and *p*-values correspond to the main effects of growing season and nutrient management strategy, and their interaction, as determined by two-way ANOVA.

**Table 8:** Results of repeated-measures ANOVA evaluating the effects of growing season, nutrient management strategy and sampling date on soil nitrite + nitrate N content in the 0–25 cm (A) and 25–50 cm (B) soil layers.

Source of Variation	df	F	*p*-Value
**(A) 0–25 cm**			
**Between-subject effects**			
Growing season	1	1.469	0.243
Nutrient management strategy	3	9.217	0.001
Growing season × Nutrient management strategy	3	15.401	<0.001
**Within-subject effects**			
Sampling date	2	93.716	<0.001
Sampling date × Growing season	2	0.220	0.804
Sampling date × Nutrient management strategy	6	1.664	0.162
Sampling date × Growing season × Nutrient management strategy	6	3.448	0.010
**(B) 25–50 cm**			
**Between-subject effects**			
Growing season	1	0.573	0.460
Nutrient management strategy	3	3.555	0.038
Growing season × Nutrient management strategy	3	6.385	0.005
**Within-subject effects**			
Sampling date	2	54.929	<0.001
Sampling date × Growing season	2	6.691	0.004
Sampling date × Nutrient management strategy	6	11.884	<0.001
Sampling date × Growing season × Nutrient management strategy	6	5.538	0.001

Soil nitrite + nitrate N content was determined at three sampling dates (following green manure incorporation, maize sowing, and maize harvest). Sampling date was treated as the within-subject factor, whereas growing season and nutrient management strategy were treated as between-subject factors. Separate repeated-measures ANOVAs were performed for the 0–25 and 25–50 cm soil depths. Because the assumption of sphericity was met (Mauchly’s test, *p* > 0.05), results are presented using the sphericity-assumed model.

## Data Availability

Data are contained within the article or Supplementary Materials: The original contributions presented in this study are included in the article/Supplementary Materials. Further inquiries can be directed to the corresponding author.

## Supplementary Materials

The following supporting information can be downloaded at: https://www.mdpi.com/article/10.3390/plants15162424/s1, Table S1. Detailed Type III ANOVA results for maize plant height and grain yield.
