# Supplementary material for: Soil Mineral Nitrogen Dynamics and Yield Stability in Maize Under Variable Precipitation: Comparing Leguminous Green Manures with Mineral Nitrogen Fertilization in a Two-Year Pilot Study
Source: Plants (Basel). 2026 Aug 8;15(16):2424. doi: 10.3390/plants15162424 (PMC13516188; doi:10.3390/plants15162424)
Supplement: Supplementary file 1 [file plants-15-02424-s001.zip › plants-4438753-supplementary.pdf]

Supplementary Materials

Supplementary Table S1. Detailed Type III ANOVA results for maize plant height and grain yield

(A) Maize plant height

Source of variation	Type III sum of squares	df	Mean square	F	P-value	Partial η^2
**Growing season**	154297.840	1	154297.840	1444.915	<0.001	0.984
**Nutrient management strategy**	2188.179	3	729.393	6.830	0.002	0.461
**Growing season × Nutrient management strategy**	1984.637	3	661.546	6.195	0.003	0.436
**Error**	2562.884	24	106.787	–	–	–

(B) Maize grain yield

Source of variation	Type III sum of squares	df	Mean square	F	P-value	Partial η^2
**Growing season**	540.562	1	540.562	967.557	<0.001	0.976
**Nutrient management strategy**	28.836	3	9.612	17.205	<0.001	0.683
**Growing season × Nutrient management strategy**	19.483	3	6.494	11.624	<0.001	0.592
**Error**	13.408	24	0.559	–	–	–

Type III tests of between-subject effects are presented for the two-way ANOVA evaluating the effects of growing season and nutrient management strategy.
